# The m^6^A methylome of SARS-CoV-2 in host cells

**DOI:** 10.1038/s41422-020-00465-7

**Published:** 2021-01-28

**Authors:** Jun’e Liu, Yan-Peng Xu, Kai Li, Qing Ye, Hang-Yu Zhou, Hanxiao Sun, Xiaoyu Li, Liu Yu, Yong-Qiang Deng, Rui-Ting Li, Meng-Li Cheng, Bo He, Jia Zhou, Xiao-Feng Li, Aiping Wu, Chengqi Yi, Cheng-Feng Qin

**Affiliations:** 1 State Key Laboratory of Protein and Plant Gene Research, School of Life Sciences, Peking University, Beijing, 100871 China; 2 Beijing Advanced Innovation Center for Genomics (ICG), Peking University, Beijing, 100871 China; 3 Biomedical Pioneering Innovation Center, Ministry of Education Key Laboratory of Cell Proliferation and Differentiation, Beijing, 100871 China; 4 State Key Laboratory of Pathogen and Biosecurity, Beijing Institute of Microbiology and Epidemiology, Academy of Military Medical Sciences, Beijing, 100071 China; 5 Academy for Advanced Interdisciplinary Studies, Peking University, Beijing, 100871 China; 6 Peking-Tsinghua Center for Life Sciences, Peking University, Beijing, 100871 China; 7 Suzhou Institute of System Medicine, Chinese Academy of Medical Sciences & Peking Union Medical College, Suzhou, Jiangsu 215000 China; 8 Department of Chemical Biology and Synthetic and Functional Biomolecules Center, College of Chemistry and Molecular Engineering, Peking University, Beijing, 100871 China

**Keywords:** RNA modification, Mechanisms of disease

## Abstract

The newly identified Severe Acute Respiratory Syndrome Coronavirus 2 (SARS-CoV-2) has resulted in a global health emergency because of its rapid spread and high mortality. The molecular mechanism of interaction between host and viral genomic RNA is yet unclear. We demonstrate herein that SARS-CoV-2 genomic RNA, as well as the negative-sense RNA, is dynamically *N*^6^-methyladenosine (m^6^A)-modified in human and monkey cells. Combined RIP-seq and miCLIP analyses identified a total of 8 m^6^A sites at single-base resolution in the genome. Especially, epidemic strains with mutations at these identified m^6^A sites have emerged worldwide, and formed a unique cluster in the US as indicated by phylogenetic analysis. Further functional experiments showed that m^6^A methylation negatively regulates SARS-CoV-2 infection. SARS-CoV-2 infection also triggered a global increase in host m^6^A methylome, exhibiting altered localization and motifs of m^6^A methylation in mRNAs. Altogether, our results identify m^6^A as a dynamic epitranscriptomic mark mediating the virus–host interaction.

## Introduction

Since December 2019, the disease named as the Coronavirus Disease 2019 (COVID-19) has rapidly spread throughout the world and become a pandemic. As of 21 December 2020, there have been 75,704,857 confirmed cases of COVID-19, including 1,690,061 deaths, reported to WHO. COVID-19 is caused by a novel coronavirus named Severe Acute Respiratory Syndrome coronavirus 2 (SARS-CoV-2). While vaccines and antiviral drugs are under development to prevent virus infection and treat the disease, little is known about the interaction between the virus and host.

Coronaviruses are enveloped RNA viruses that are broadly distributed among humans, other mammals and birds, causing acute and persistent infections. All coronaviruses can be divided into four genera: alphacoronaviruses, betacoronaviruses, gammacoronaviruses, and deltacoronaviruses.^1^ The emerged SARS-CoV-2 belongs to betacoronaviruses, together with the other two highly pathogenic human coronaviruses, SARS and Middle East Respiratory Syndrome Coronavirus (MERS-CoV).^2^ SARS-CoV-2 has a single-stranded, positive-sense genomic RNA of approximately 30 kb in length.^3^ Following the entry of SARS-CoV-2, viral genome is released and translated into viral replicase polyproteins, which are then processed by host viral proteinases. The full-length negative-sense template is synthesized from the positive-sense genomic RNA and made as a template for progeny viral RNA synthesis. Subgenomic negative-sense templates are also synthesized from discontinuous transcription and serve as templates for mRNA synthesis. Like other coronaviruses, the genome of SARS-CoV-2 has a standard eukaryotic 5′-terminal cap structure and a 3′ polyadenylate tail.^4^ The cap structure and epitranscriptomic modification like 2′-O-MTase methylation have been demonstrated to stabilize the coronavirus RNA by blocking degradation via the 5′-3′ exoribonuclease and evade the recognition of host RNA sensors or resist the interferon (IFN)-mediated antiviral response.^5,6^ However, whether or not any internal modification exits in the viral genome of SARS-CoV-2 remains unknown.

More than 100 types of post-transcriptional RNA modifications have been characterized thus far.^7^ They are mostly present in abundant ribosome RNA (rRNA) and transfer RNA (tRNA), with a dozen of modifications present in messenger RNA (mRNA). The *N*^6^-methyladenosine (m^6^A), firstly discovered in 1974 by Desrosiers et al., is the most abundant internal modification of mRNA and lncRNA in mammalian cells.^8–11^ m^6^A on mRNA is commonly found within a consensus motif DRm^6^ACH (where D represents A, G or U; R represents G or A; H represents A, C, or U), and is reversible and dynamically regulated by its “writers” and “erasers”. m^6^A is catalyzed by a methyltransferase complex containing at least of the core catalytic heterodimer (METTL3 and METTL14), a splicing factor (WTAP) and other cofactors including KIAA1429, HAKAI, ZC3H13 and RBM15/15B.^12–14^ m^6^A is the first discovered reversible mRNA modification and is demethylated via FTO and ALKBH5.^15,16^ m^6^A is widely distributed along mRNA and enriched around stop codons.^17,18^ Different types of reader proteins can specifically recognize m^6^A-containing RNAs and play important roles in regulating the fate of m^6^A-marked mRNA.^19^ For instance, the YTH-domain family 2 (YTHDF2), the first reported m^6^A reader protein, binds to m^6^A-containing mRNAs via its carboxy-terminal YTH domain so as to promote mRNA degradation.^20^ So far, literature has documented the pivotal roles of m^6^A in regulating various aspects of RNA metabolism, including RNA localization, splicing, stability and translation.^11,21–24^

m^6^A has also long been identified in RNA transcripts of viruses, including Rous sarcoma virus, influenza virus, simian virus 40, avian sarcoma virus and adenovirus;^25–28^ yet its roles in viral life cycle regulation still remain unclear. Recent studies have demonstrated that m^6^A modification in HIV and ZIKV viral RNA can regulate virus gene expression and influence viral replication.^29–33^ However, little is known about the distribution, function and regulatory mechanism of m^6^A in coronaviruses including the newly identified SARS-CoV-2.

In this study, we profiled the m^6^A methylome of SARS-CoV-2 in human and monkey cells, and demonstrated that m^6^A was widely distributed in both positive-sense and negative-sense SARS-CoV-2 RNA. Particularly, hundreds of epidemic strains with mutations disrupting the m^6^A motif have emerged worldwide. Viral infection triggered relocation of key modification enzymes from the nucleus to the cytoplasm, with m^6^A writers METTL3/14 and eraser ALKBH5 negatively and positively regulates SARS-CoV-2 replication, respectively. SARS-CoV-2 replication is sensitive to the m^6^A reader YTHDF2 as well. We also found that SARS-CoV-2 infection alters the host m^6^A methylome, suggesting that m^6^A is involved in the host–virus interaction. Altogether, our results report the host and viral m^6^A methylome during SARS-CoV-2 infection, highlighting the potential roles of m^6^A during SARS-CoV-2 transmission and pathogenesis.

## Results

### m^6^A methylome in positive-sense genomic RNA of SARS-CoV-2

**Fig. 1:**
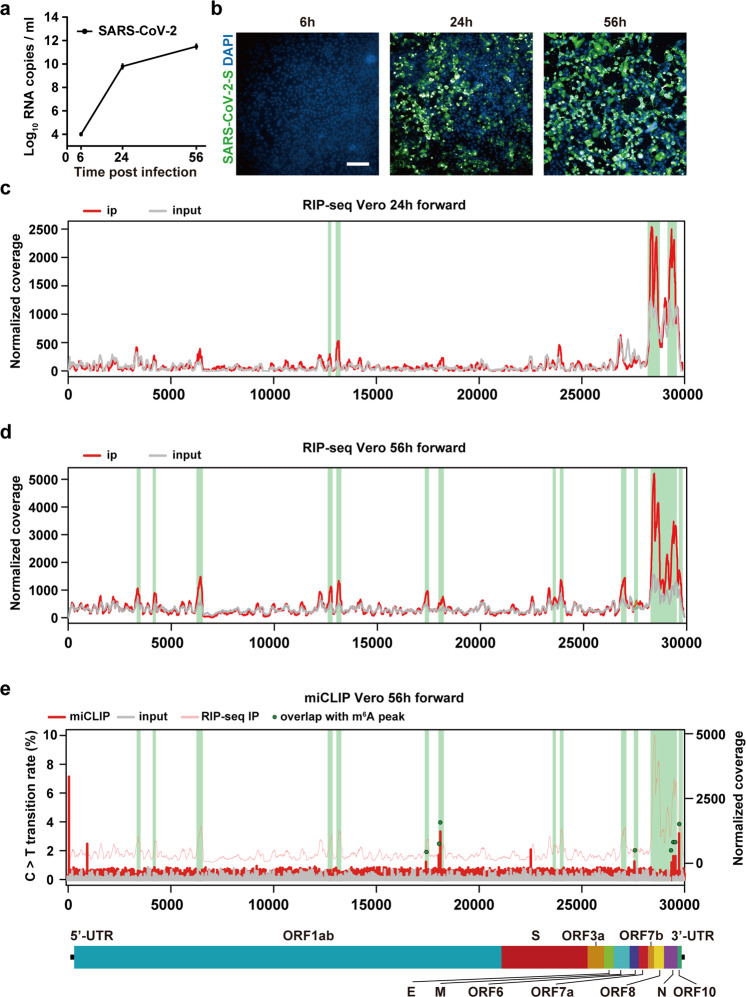
Landscape of m^6^A methylome in positive-sense genomic RNA of SARS-CoV-2. **a** Vero cells were infected with SARS-CoV-2. Supernatant was harvested 6, 24, 56 h later for quantification of SARS-CoV-2 RNA by qRT-PCR. *n* = 3. **b** Representative fields of SARS-CoV-2-infected Vero cells (S protein, green) and nuclei (DAPI, blue) at different time points including 6, 24 and 56 hpi. Scale bar, 20 µm. **c**, **d** Refined RIP-seq of SARS-CoV-2 RNA harvested from Vero cells at 24 hpi (**c**) and 56 hpi (**d**) showing the distribution of m^6^A reads mapped to SARS-CoV-2 genome (red line). The gray line represents the baseline signal from input samples, and green rectangles along the *x*-axis show m^6^A peaks. Data are representative of *n* = 2 determinations. **e** miCLIP revealed m^6^A sites at single-base resolution across SARS-CoV-2 genome. The ratio of C-to-T transitions of input samples and immunoprecipitation samples of miCLIP, and the distribution of SARS-CoV-2 reads of immunoprecipitation samples by RIP-seq are represented by gray line, red line and pink line, respectively. The green dots show m^6^A sites that locate within the identified m^6^A peaks and are identified in both two biological replicates of miCLIP. A schematic diagram of the SARS-CoV-2 genome is shown below to indicate the location of the m^6^A-enriched sequences.

To investigate whether the genomic RNA of SARS-CoV-2 was m^6^A methylated, African green monkey kidney cell line Vero and human hepatocarcinoma cell line Huh7 that are susceptive to SARS-CoV-2 were used in this study. As expected, SARS-CoV-2 viral RNAs multiplicated rapidly in Vero cells following SARS-CoV-2 infection (Fig. 1a; Supplementary information, Table S1). Immunofluorescence assay with SARS-CoV-2 S-specific monoclonal antibody showed that the percentage of virus-infected cells increased as the infection time extended, and reached to nearly 100% at 56 hours post infection (hpi) (Fig. 1b). The topology of m^6^A methylome of SARS-CoV-2 was initially determined by a refined RNA immunoprecipitation followed by high-throughput sequencing (RIP-seq) assay. Total RNAs extracted from Vero cell supernatant were fragmented into 100–200 nt and m^6^A-marked transcripts were enriched by immunoprecipitation with an m^6^A antibody (see Materials and methods). The SARS-CoV-2 RNAs were sequenced at sufficient depth for input (~1100–2500× and ~43,000–60,000× for 24 hpi and 56 hpi, respectively) and immunoprecipitated (~40–80× and ~1400–1900× for 24 hpi and 56 hpi, respectively) samples to detect potential m^6^A signals. As a positive control, the known m^6^A site at position 4190 of 28S rRNA was successfully enriched (Supplementary information, Fig. S1a); and a high correlation (0.9947) was observed between two biological replicates (Supplementary information, Fig. S1b), suggesting good reproducibility of our approach. There were four confident m^6^A peaks at the SARS-CoV-2 genome at 24 hpi (Fig. 1c), whereas nine additional confident m^6^A peaks were detected spanning the full-length genomic RNA of SARS-CoV-2 at 56 hpi (Fig. 1d; Supplementary information, Table S2), suggesting m^6^A modification occurred at the late stage of infection. All the m^6^A peaks identified in Vero cells were validated by m^6^A-IP-qPCR (Supplementary information, Fig. S1c and Table S1), and the m^6^A intensity of SARS-CoV-2 at 56 h is higher than that of 24 h (Supplementary information, Fig. S1d). Further validation in SARS-CoV-2-infected Huh7 cells with RIP-Seq also detected 6 confident m^6^A peaks in SARS-CoV-2 genomic RNA at 120 hpi (Supplementary information, Fig. S1e, f and Table S2), all of which overlapped with m^6^A peaks identified in SARS-CoV-2-infected Vero cells at 56 hpi. These data demonstrate that SARS-CoV-2 RNA is gradually m^6^A methylated during the infection of SARS-CoV-2 in host cells.

### Identification of the precise m^6^A sites in SARS-CoV-2 genomic RNA

**Fig. 2:**
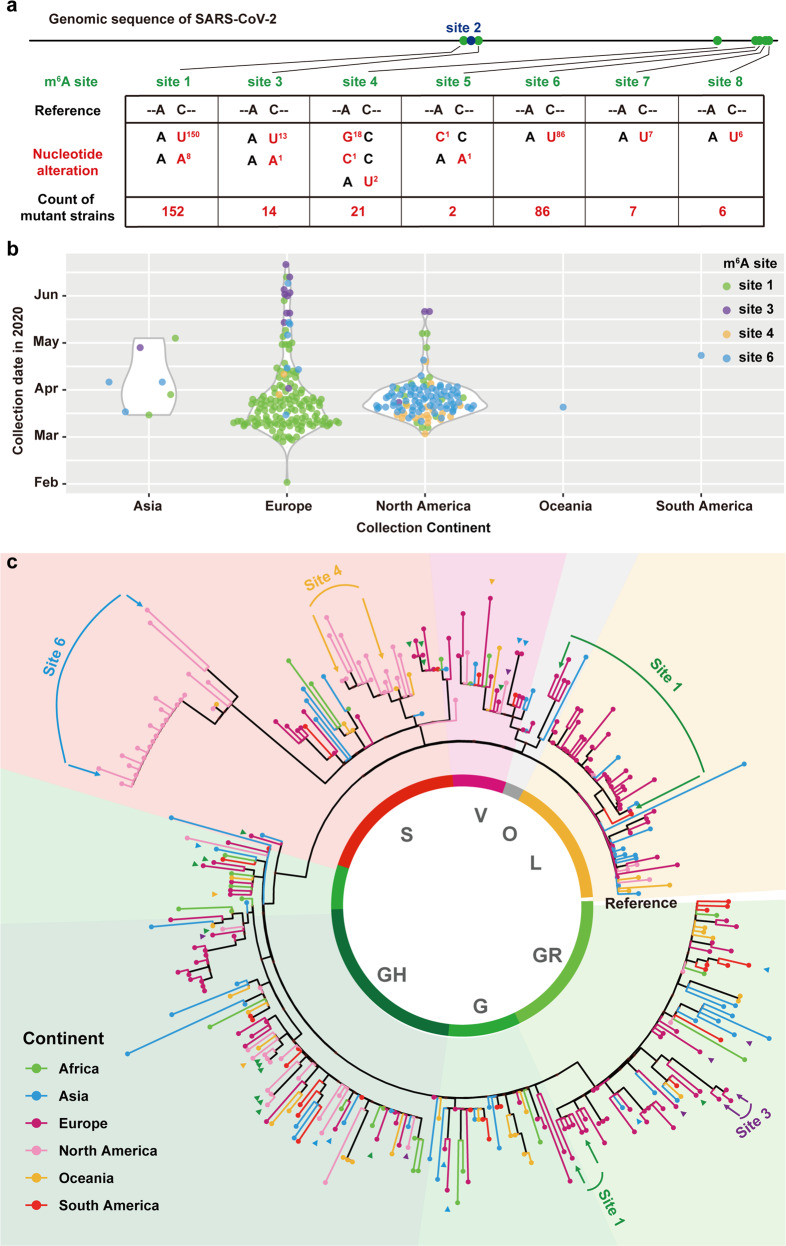
m^6^A substitution among diverse SARS-CoV-2 isolates. **a** Sequence comparison is performed by python with Biopython package. The EPI_ISL_424359 (blue) is set as the reference sequence. The contemporary SARS-CoV-2 strains used in this study are labeled in black and red. Variations are presented in accordance with their locations in viral genome. **b** The sample distribution of 289 strains with mutations at m^6^A modification site 1, 3, 4 and 6, each point represents one sample and plotted by R with ggplot2 package. Most of the mutant strains were found distributed in Europe and North America. **c** The maximum likelihood phylogenetic tree estimated using IQ-TREE. The final result is visualized by ggtree. Conserved nucleotide changes are inferred using the EPI_ISL_424359 strain as the reference strain. Virus sequences selected from 7 GISAID clade (L, S, G, GH, GR, O, V) and the continent information is shown in different colors. The positions of mutant strains of m^6^A sites are indicated with arrows colored by green (Site 1), purple (Site 3), yellow (Site 4) and blue (Site 6). The details of the tree are shown in Supplementary information, Table S4.

To identify the exact m^6^A modification at single-base resolution, a modified m^6^A individual-nucleotide-resolution cross-linking and immunoprecipitation (miCLIP) assay^34^ was performed in SARS-CoV-2-infected Vero cells. We also achieved high sequencing depth (~2,450,000× and ~1,600,000× for immunoprecipitated and input samples, respectively) to improve the confidence of miCLIP experiments. For instance, the C-to-T transition rate of m^6^A sites is significantly higher than that of background (Supplementary information, Fig. S2a), and the distribution of host m^6^A sites and the consensus motif of m^6^A sites in Vero cells identified by miCLIP resembled previous reports (Supplementary information, Fig. S2b and c). 12 and 11 single-based m^6^A sites were identified in each biological replicate, respectively (Fig. 1e; Supplementary information, Fig. S2d), with 11 shared m^6^A sites. Among them, 8 m^6^A sites overlapped with m^6^A peaks identified by RIP-seq (marked by green points in Fig. 2a); hence, they were used for further analysis.

We then analyzed the m^6^A sites according to the schematic diagram of the reference genome. We found 3 m^6^A sites in ORF1ab, 1 m^6^A site in ORF 7a, 3 m^6^A sites in N, and 1 m^6^A site in ORF 10 (Supplementary information, Table S3). Thus, it appeared that m^6^A modification preferred to occur more frequently towards the 3′ end of the viral genome.

### SARS-CoV-2 epidemic strains contain mutations at m^6^A sites

Consistent with the previous finding that m^6^A peaks are enriched for SNPs across human tissues,^35^ the m^6^A sites of SARS-CoV-2 are also enriched for SNPs: as the distance to the m^6^A sites increases, the number of SNPs decreases (Supplementary information, Fig. S3), suggesting m^6^A is relatively less conserved among the present SARS-CoV-2 isolates. However, during the global transmission of SARS-CoV-2, a panel of mutations that potentially impacted viral transmission and pathogenicity have been recently identified.^36^ To monitor the substitutions at m^6^A sites, all available full-length (length > 29,000 bp) SARS-CoV-2 genome sequences with complete meta data in GISAID till July 16th were used for analysis. After removing duplicate and low-quality sequences (>5% NNNNs), all of the 56,143 sequences were subjected to sequence alignment using MAFFT^37^ and analyzed by python with Biopython package.^38^ Surprisingly, a total of 288 epidemic strains containing nucleotide mutation at A or C of the core motif for m^6^A sites were identified (Fig. 2a; Supplementary information, Table S4). These mutations were expected to disrupt the m^6^A modification.^39,40^ No nucleotide mutations were identified for site 2, and for the remaining m^6^A sites, at least one mutant strain was found. The detailed epidemiological information of the genomes with mutation at m^6^A modification sites could be found in Supplementary information, Table S4. The distribution of viral strains was plotted by ggplot2^41^ (Fig. 2b). It could be found that the mutant strains in sites 1 and 3 were predominantly isolated in the Europe, while the mutant strains in sites 4 and 6 were mainly isolated in North America (Fig. 2b).

To further evaluate the potential impact of these unique mutations at the identified m^6^A sites from an evolutionary perspective, a maximum likelihood phylogenetic tree with bootstrap test (replicated 1000 times) of the representative strains with and without mutations at the m^6^A modification sites was constructed using IQ-TREE.^42^ Similar to the previous finding,^43^ all epidemic strains were divided into 7 clades including L, S, G, GH, GR, O, and V clades (Fig. 2c). Most of the identified m^6^A mutant strains locates dispersedly in different clades, while mutant strains at site 6 form a unique clade within clade S in the phylogenetic tree, highlighting the potential evolutionary role of m^6^A on SARS-CoV-2 transmission and epidemiology.

### Negative-sense SARS-CoV-2 RNA is modified by m^6^A

**Fig. 3:**
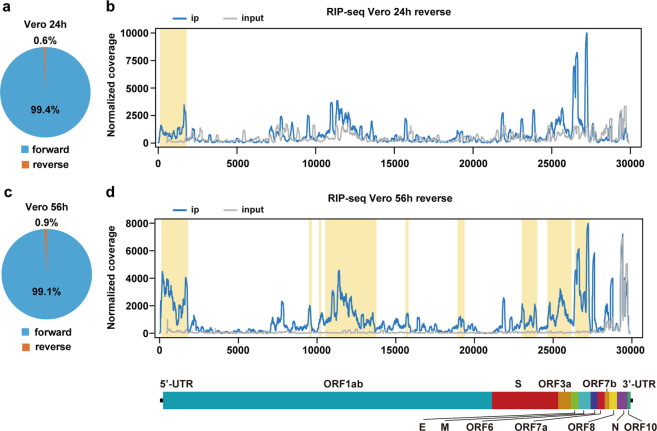
Topology of m^6^A methylome in negative-sense RNA of SARS-CoV-2. **a** The ratio of positive-sense and negative-sense RNA of SARS-CoV-2 harvested from Vero cells at 24 hpi. **b** Map of m^6^A peaks identified in the negative-sense RNA of SARS-CoV-2 isolated from SARS-CoV-2-infected Vero cells at 24 hpi by RIP-seq. Read coverage is normalized to the total number of reads mapping to the viral negative-sense RNA for each experiment. The distribution of m^6^A reads mapped to the SARS-CoV-2 negative-sense RNA is shown as blue line and the baseline signal from the RNA-seq signal of input samples is shown as a gray line. m^6^A peaks are shown as yellow rectangles along the *x*-axis. **c** The proportion of positive-sense and negative-sense RNA of SARS-CoV-2-infected Vero cells at 56 hpi. **d** m^6^A reads mapped to the SARS-CoV-2 negative-sense RNA extracted from SARS-CoV-2-infected Vero cells at 56 hpi. Data are representative of *n* = 2 determinations. The blue line shows the distribution of m^6^A reads mapped to the SARS-CoV-2 negative-sense RNA and the gray line shows the baseline signal of input samples. m^6^A peaks are shown as yellow rectangles along the *x*-axis.

Besides the positive-sense viral genome, negative-sense RNA intermediates are also of great importance in serving as the templates for the synthesis of positive-sense genomic RNA and subgenomic RNAs.^44^ In our RIP-seq data, negative-sense RNA of SARS-CoV-2 accounts for less than 1% sequencing reads of positive-sense genomic RNA (Fig. 3a), consistent with its intermediary role. Because of the directionality of the template-switching reaction we adopted,^45^ we preserved the strand orientation of the original RNA, allowing us to distinguish m^6^A signals in positive-sense RNA and negative-sense RNA. We were able to identify 1 m^6^A peak at the 5′ end of the negative-sense RNA harvested from SARS-CoV-2-infected Vero cell at 24 hpi (Fig. 3b). Similar proportion of negative-sense of SARS-CoV-2 RNA was found in total sequencing reads at 56 hpi, and additional 8 m^6^A signals were identified in the negative-sense SARS-CoV-2 RNA (Fig. 3c, d), demonstrating that m^6^A is prevalent in negative-sense RNA as well. Due to the limited coverage of the negative-sense RNA, we were not able to identify high-confidence m^6^A sites using miCLIP. Nevertheless, our results clearly demonstrated that the negative-sense RNA of SARS-CoV-2 is also dynamically m^6^A methylated during viral infection.

### m^6^A RNA methylation negatively regulates the SARS-CoV-2 life cycle

We further observed subcellular localization of m^6^A writers and erasers in response to SARS-CoV-2 infection. As expected, methyltransferase METTL14 and demethylase ALKBH5 were normally expressed in nucleus of the uninfected Huh7 cells (Supplementary information, Fig. S4); However, in the SARS-CoV-2-infected Huh7 cells, abundant METTL14 and ALKBH5 were relocated into cytoplasm, where coronavirus genomic RNA replication occurs.

**Fig. 4:**
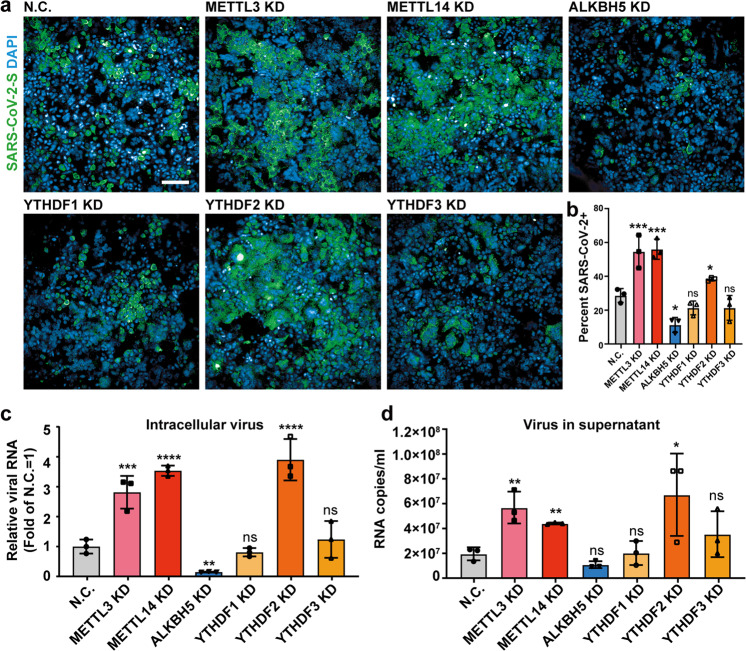
m^6^A inhibits SARS-CoV-2 replication. **a** Immunofluorescence images of SARS-CoV-2-infected Huh7 cells after m^6^A methyltransferases (METTL3/14), demethylase (ALKBH5) and reader proteins (YTHDF1, YTHDF2 and YTHDF3) were knocked down, respectively. Huh7 cells were treated with siRNAs targeting METTL3/14, and ALKBH5, meanwhile a non-targeting siRNA as the negative control and then were infected by SARS-CoV-2 at an MOI of 0.05 for 72 h. Specific antibodies recognizing the SARS-CoV-2 S protein (green) were used; DNA was stained with DAPI (blue). Scale bar, 20 µm. **b** Percentage of viral S protein-positive cells as treated in **a**. *n* = 3. Statistical significance of the difference was determined by Student’s unpaired *t*-test. *P < 0.05; ***P < 0.001; ns, non-significant. **c** Relative SARS-CoV-2 RNA copies in siRNA-treated cells quantified by qRT-PCR at 72 hpi. RNA was normalized with GAPDH. *n* = 3. All data are the means ± SD of the indicated number of replicates. Statistical significance of the difference was determined by Student’s unpaired *t*-test. **P < 0.01; ***P < 0.001; ****P < 0.0001; ns, non-significant. **d** The released SARS-CoV-2 RNA in siRNA-treated supernatants was quantified by qRT-PCR at 72 hpi. *n* = 3. All data are the means ± SD of the indicated number of replicates. Statistical significance of the difference was determined by Student’s unpaired *t*-test. *P < 0.05; **P < 0.01; ns, non-significant.

To further investigate whether m^6^A regulates SARS-CoV-2 infection, we knocked down known m^6^A writers, erasers and readers by small interfering RNA (siRNA) in Huh7 cells. Knockdown efficiency was assessed by western blot and quantitative PCR (RT-qPCR) analyses (Supplementary information, Fig. S5a, b and Table S1). The KD cells were then infected with SARS-CoV-2 at a multiplicity of infection (MOI) of 0.05. The immunofluorescence images and statistical results at 72 hpi showed that viral replication and the percentage of SARS-CoV-2-positive cells increased significantly after METTL3 and METTL14 were knocked down; conversely, viral replication was decreased after ALKBH5 was knocked down (Fig. 4b, c). Meanwhile, knocking down YTHDF2, but not YTHDF1 and YTHDF3, was conducive to the viral infection and replication (Fig. 4b, c). In addition, relative viral RNA growth in cells and the viral RNA copies released in supernatant were also measured, which demonstrated the same tendency of SARS-CoV-2 infection affected by m^6^A-related protein depletion (Fig. 4d, e). Thus, modulation of the m^6^A RNA methylome by host factors profoundly influences viral replication, with m^6^A imposing a negative regulatory role on SARS-CoV-2 infection.

### SARS-CoV-2 infection influences m^6^A methylome of host cell

**Fig. 5:**
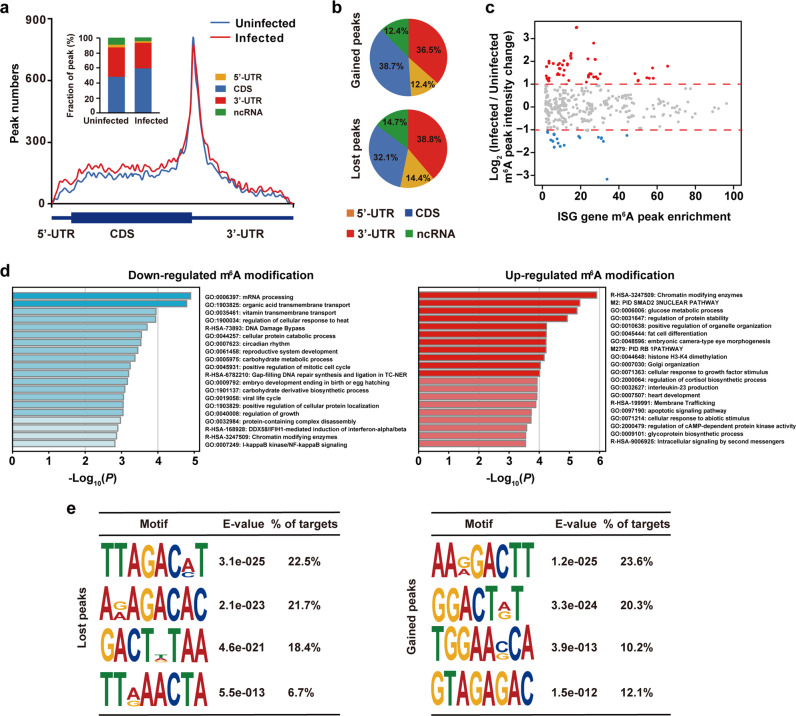
SARS-CoV-2 infection influences m^6^A methylome of Huh7 cell transcripts. **a** Distribution of the enriched m^6^A peaks in uninfected (blue line) and SARS-CoV-2-infected Huh7 cells (red line) analyzed along the RNA segments. Each segment was normalized according to its average length in Refseq annotation. Barplot shows the percentage of m^6^A peaks in the indicated regions. **b** Charts showing the distribution of gained (top) and lost m^6^A peaks (bottom) in the 5′ UTR (orange), CDS (blue), 3′ UTR (red) and ncRNA (green) of host cell RNA transcripts. Huh7 cells were uninfected or SARS-CoV-2 infected, and m^6^A peaks in total cellular RNA were analyzed at 120 h after infection. Representative of *n* = 2 determinations. **c** MA plot showing changes in m^6^A peak intensity of interferon-stimulated genes before and after SARS-CoV-2 infection. Upon SARS-CoV-2 infection, interferon-stimulated genes with no obvious changes in m^6^A peak intensity, higher m^6^A peak intensity and lower m^6^A peak intensity are represented by gray dots, red dots and blue dots, respectively. **d** GO enrichment analyses of genes with downregulated m^6^A peaks (left) and upregulated m^6^A peaks (right). **e** Motif analysis to identify consensus sequences for lost m^6^A peaks (left) and gained m^6^A peaks (right). The top four motifs for each are shown.

Because m^6^A modification machineries exhibit re-localization in response to viral infection (Fig. 4a), we hypothesized that viral infection may impact the m^6^A methylome of the host cells. Thus, we detected m^6^A abundance in uninfected and SARS-CoV-2-infected Vero and Huh7 cells, and found that their m^6^A abundance increased upon SARS-CoV-2 infection (Supplementary information, Fig. S6a and b). We then sought to investigate whether and how SARS-CoV-2 infection would influence the distribution of m^6^A on cellular transcripts. We adopted the refined RIP-seq experiments to cellular RNA extracted from the uninfected and SARS-CoV-2-infected Huh7 cells at 120 hpi, respectively. We found that SARS-CoV-2 infection led to an increased m^6^A level in the coding sequence (CDS) regions and a concomitant decreased m^6^A level in the 3′ UTR (Fig. 5a). We further defined m^6^A peaks uniquely identified in SARS-CoV-2-infected Huh7 cells as gained m^6^A signals while m^6^A peaks only found in uninfected Huh7 cells as lost m^6^A signals. SARS-CoV-2 infection triggers an increase of m^6^A signals in host, with 8967 gained peaks and 3845 lost peaks respectively (Supplementary information, Table S5). Consistent with above finding, we found that the overall m^6^A intensity significantly increased in SARS-CoV-2-infected Huh7 cells compared with that of uninfected Huh7 cells (Supplementary information, Fig. S6c), suggesting that viral infection altered the host m^6^A methylome. Post SARS-CoV-2 infection, the gained m^6^A modifications prefer to locate in CDS region in comparison to lost m^6^A signals (Fig. 5b). We further explored the relationship between m^6^A signals and expression level, and found that m^6^A changes do not correlate with expression level changes of host transcripts in the global level (Supplementary information, Fig. S6d). Nevertheless, we observed that more interferon-stimulated genes undergo increased m^6^A methylation compared to those exhibiting decreased m^6^A methylation (Fig. 5c). Moreover, we found that the expression level of interferon-stimulated genes was not significantly changed between the uninfected and SARS-CoV-2-infected Huh7 cells (Supplementary information, Fig. S6e), indicating that the increased m^6^A level in the interferon-stimulated genes was not due to an RNA expression level changes but instead was a host response to viral infection at post-transcription level. Moreover, Gene ontology (GO) enrichment analysis of genes with upregulated m^6^A modification (fold change > 2) showed that membrane trafficking categories and apoptotic signaling pathway were enriched, while viral life cycle was enriched in the genes with downregulated m^6^A signals (fold change > 2) (Fig. 5d). Additionally, motif analysis of m^6^A signals in SARS-CoV-2-infected Huh7 cells was performed to explore if there was any change in the consensus motif post viral infection. We found that the motif usage showed slight changes on the overall level (Supplementary information, Fig. S6f and g). To further investigate the gained and lost m^6^A peaks, we found that the gained m^6^A has a “GGACH” motif while lost m^6^A signals are residing in the “AGACH” context (Fig. 5e), suggesting that the substrate specificity of m^6^A modification machineries may vary upon SARS-CoV-2 infection.

## Discussion

Despite that a comprehensive understanding of SARS-CoV-2 is pivotal, post-transcriptional modification of SARS-CoV-2 was unclear. In this study, we provide the first transcriptome-wide characterization of m^6^A methylome of SARS-CoV-2. Interestingly, we find that m^6^A is widely distributed and dynamically regulated in the positive-sense genome and negative-sense RNA intermediates. Meanwhile, hundreds of epidemic strains with mutations disrupting the m^6^A motif were identified as well. We showed a viral suppressive role of m^6^A as m^6^A methyltransferases and demethylase are involved in viral life cycle regulation. Moreover, YTHDF2, which has a documented role to decay m^6^A-marked transcripts,^20^ negatively regulates SARS-CoV-2 replication. Furthermore, we uncover that host m^6^A methylome including m^6^A location and methylation motifs is changed post SARS-CoV-2 infection. Collectively, our study reveals that m^6^A modifications are widespread and dynamically regulated epitranscriptomic marks in SARS-CoV-2.

m^6^A and its reader proteins have diverse roles during viral infection; yet to date there is no report about the roles of m^6^A in the life cycle regulation of coronavirus. For *Flaviviridae* family, m^6^A is found in ZIKV, dengue virus, West Nile virus, yellow fever virus and hepatitis C virus (HCV), and plays a negative role in ZIKV and HCV infection.^29,32^ While for HIV-1, m^6^A in its viral genome has been reported to either enhance or inhibit HIV-1 replication,^30,31,33^ partly due to different m^6^A sites and reader proteins interrogated by the studies. m^6^A readers, which play many important biological roles including RNA stability, decay, transport and protein translation, have distinct effects on the life cycles of different viruses. It is reported that m^6^A readers binding to m^6^A can mark human metapneumovirus (HMPV) RNA and circRNA as self-RNA to protect from immune response.^46,47^ However, another work indicates that reader proteins suppress HIV-1 infection and viral production.^48^ We uncover in this study that m^6^A acts as a negative regulator for SARS-CoV-2, adding to our knowledge of epitranscriptomic regulation in coronavirus. However, further investigations are needed to explore whether it is the viral m^6^A or the host m^6^A that inhibits SARS-CoV-2 replication, as m^6^A modifications of host cells are important for antiviral response.^49^

Epidemiological implications of the m^6^A sites to the SARS-CoV-2 were pregnant. Through the completed blast, there were 288 mutant strains whose nucleotide mutated at the identified m^6^A sites in 56,143 SARS-CoV-2 sequences. Most of the recorded mutants distributed relatively concentrated in different continents across different stages. Mutants at site 1 emerged at the early stage of pandemic, and followed by the emergence of other mutants. Of particular note, the mutant strains at site 6 formed a unique cluster within Clade S (Fig. 2c). All these strains contained C29451T mutation, and most of them were isolated in 4 different states of USA (23/24) from 13th March to 9th April. Additionally, this mutation will lead to the T393I mutation in N protein, the biological outcome of this unique mutation deserves further investigation.

We revealed that the m^6^A is present in the negative-sense RNA of SARS-CoV-2, demonstrating for the first time that viral RNA intermediates are also subjected to epitranscriptomic regulation. It is tempting to speculate that m^6^A on the negative-sense RNA may function as a new layer of regulation of SARS-CoV-2 replication. Because the negative-sense RNA accounts for less than 1% sequencing reads of the positive-sense RNA, m^6^A-mediated decay of the key RNA intermediary for viral replication and subgenomic RNA synthesis could represent an attractive approach by the host cells to counteract viral infection.

We also found that SARS-CoV-2 infection leads to dynamic change of host m^6^A methylome. Upon viral infection, an increase of m^6^A methylome was found for both the viral genome and host mRNA. This is at least in part due to a re-localization of METTL14 and ALKBH5 into the cytoplasm where SARS-CoV-2 replication and transcription occurs. In contrast, no obvious redistribution of the enzymes was found upon ZIKV infection.^29^ Along this line, the altered m^6^A motifs are also different for the two viruses. Given the previous report that elongation-promoting effect of CDS methylation mediated by m^6^A requires the RNA helicase-containing m^6^A reader YTHDC2,^50^ and the finding that gained m^6^A signals prefer to locate in CDS region post SARS-CoV-2 infection (Fig. 5b), further investigations of the function of these increased m^6^A signals in CDS region are needed in the future (Fig. 5b). Altogether, viral infection of SARS-CoV-2 triggered the reprogramming of m^6^A methylome in host cells.

Our finding that m^6^A acts as a negative regulator of SARS-CoV-2 replication provides potential new strategies for the development of vaccine and antiviral drugs. On one hand, attenuated vaccine strains could be designed by increasing the m^6^A modification level via reverse genetic approach. Using miCLIP, we identified several candidate m^6^A sites at base resolution; it remains to be determined whether a subset or all of them function in regulating the viral infection. Nevertheless, key m^6^A sites could be characterized and utilized in the design of attenuated vaccine strains. On the other hand, the m^6^A modification machineries could provide new targets for antiviral therapies. For instance, small molecule drugs modulating the catalytic activities of the enzymes could regulate virus infection and potentially serve as antiviral approaches. While no activator of m^6^A methyltransferases have been reported so far, literature has documented multiple small molecule inhibitors of the demethylase. For instance, N-oxalylglycine (NOG), 2,4-pyridinedicarboxylate (2,4-PDCA), IOX3 and imidazobenzoxazin-5-thione MV1035 could serve as inhibitors to reduce the activity of the ALKBH5.^51,52^

In summary, our study reveals that m^6^A RNA modification is prevalent in SARS-CoV-2, and highlights an epitranscriptomic layer of regulation for the life cycle of SARS-CoV-2 and its potential impact on SARS-CoV-2 transmission and pathogenicity. Such knowledge could promote the development of new antiviral drugs based on the post-transcriptional m^6^A modification, and pave the way for an attenuated vaccine strain design by manipulating the m^6^A mark.

## Materials and methods

### Cell culture and virus sample preparation

African green monkey kidney cell line Vero (ATCC, CCL-81) and human hepatocarcinoma cell line Huh7 (JCRB, 0403) were cultured in Dulbecco’s modified eagle medium (DMEM, Thermo Fisher Scientific, 11995065) containing 10% or 15% fetal bovine serum (Gibco, 10060141), and supplemented with 100 U/mL penicillin and 100 mg/mL streptomycin (Gibco, 15140122) at 37 °C in a humidified atmosphere with 5% CO_2_. 60 pmol siRNA was transfected into the Huh7 cells to knock down the m^6^A-related components by Lipofectamine^™^ RNAiMAX Transfection Reagent (Thermo Fisher Scientific, 13778100) in Opti-MEM^™^ (Thermo Fisher Scientific, 31985088).

SARS-CoV-2 strain BetaCov/Wuhan/IME-BJ01/2020 (GWHACBB01000000) was prepared to infect different cell types. The virus was the fourth passage. Briefly, the cell culture supernatants were discarded and virus-containing medium was added to infect the cells at an MOI of 0.001 for Vero and 0.05 for Huh7. After an incubation at 37 °C for 1 h, the virus inoculum was removed and fresh DMEM containing 2% FBS was added to each well. At different time points post infection, cells were fixed with 4% PFA (paraformaldehyde) for 15 min at room temperature (RT) for the next immunostaining or were lysed by RNA and protein lysis buffer.

### RNA isolation, DNase treatment and determination

Viral or cellular RNAs were extracted using the Purelink RNA Mini Kit (Thermo Fisher Scientific, 12183025) according to the manufacturer’s instructions. DNase I (NEB, M0303L) treatment was adopted to remove DNA contamination following by phenol-chloroform isolation and ethanol precipitation treatment to remove enzyme contamination. SARS-CoV-2 genomic RNA was quantified by one step PrimeScript^TM^ RT-qPCR Kit (Takara, RR064A). The expression level of m^6^A enzymes were quantified using a one-step SYBR Green^®^ PrimeScript^™^ PLUS RT-PCR Kit (Takara, RR096A). Primers, probes and oligonucleotides were listed in Supplementary information, Table S1.

### Refined RIP-seq of SARS-CoV-2

This procedure was performed according to the recently described methods with several modifications.^35,53,54^ Three micrograms of total RNA (250 ng viral RNA and ~2750 ng HEK293T cell RNA) was fragmented into ~150-nucleotide-long fragments by magnesium RNA fragmentation buffer (NEB, E6150S). The fragmentation was stopped by RNA fragmentation stop solution followed by ethanol precipitation. Six nanograms of fragmented total RNA was used as input and remained RNA was used to perform m^6^A immunoprecipitation. Briefly, RNA was denatured at 65 °C for 5 min, followed by chilling on ice immediately. Thirty microliters of protein A magnetic beads (Thermo Fisher Scientific, 10002D) and 30 μL protein G magnetic beads (Thermo Fisher Scientific, 10004D) were mixed and washed twice by IPP buffer (10 mM Tris-HCl, pH 7.5, 150 mM NaCl, 0.1% IGEPAL CA-630) and resuspended in 500 μL of IPP buffer. The 6 μg anti-m^6^A polyclonal antibody (Millipore, ABE572) was added to the beads and incubated at 4 °C for about 6 h. Following the beads–antibody incubation, the beads were washed twice by IPP buffer and resuspended with 500 μL mixture (fragmented total RNA, 5 μL of RNasin Plus RNase Inhibitor (Promega, N2615) and 100 μL of 5× IPP buffer) and incubated at 4 °C for 2 h, rotating head over tail. The beads–antibody–RNA mixture was washed with IPP buffer, low-salt IP buffer (50 mM NaCl, 10 mM Tris-HCl, pH 7.5, 0.1% IGEPAL CA-630) and high-salt IP buffer (500 mM NaCl, 10 mM Tris-HCl, pH 7.5, 0.1% IGEPAL CA-630). After extensive washing, 6.7 mM *N*^6^-methyladenosine (Sigma, M2780) was used to elute m^6^A-marked RNA. Fragmented total RNA (Input) and immunoprecipitated m^6^A-marked RNA (IP) were then subjected to library construction using SMARTer^®^ Stranded Total RNA-Seq Kit v2-Pico Input Mammalian (Takara, 634413) according to the manufacturer’s protocol. The 5′ end sequence information of RNA and the strand orientation of the original RNA is preserved by the directionality of the template-switching reaction. Libraries for immunoprecipitated RNA were PCR-amplified for 13 cycles whereas 11 cycles were performed for input RNA. The libraries were sequenced on Illumina Hiseq X Ten with paired-end 2 × 150 bp read length. It is noted that the preserved strand orientation of the original RNA and the condition of elution allows identifying m^6^A peaks both in positive-sense genomic RNA and negative-sense RNA for SARS-CoV-2 with a high signal-to-noise (S/N) ratio.

### m^6^A-IP-qPCR-based m^6^A peak validation

All the m^6^A peaks identified in Vero cells were validated by m^6^A-IP-qPCR using a different m^6^A antibody (Abcam, ab151230) as an orthogonal evidence to the originally used Millipore m^6^A antibody in RIP-seq. The immunoprecipitated RNA enriched by the m^6^A antibody in SARS-CoV-2-infected Vero cell line was reverse transcribed using a Revert Aid First Strand cDNA Synthesis Kit (Thermo, K1622). The enrichment fold of m^6^A-marked viral RNA was detected by qPCR. The enrichment fold of immunoprecipitated versus input of each peak was calculated and normalized to negative control. Primers were listed in Supplementary information, Table S1.

### m^6^A-miCLIP-seq of SARS-CoV-2

m^6^A methylome of SARS-CoV-2 was profiled at single-base resolution following previously reported methods with some modifications.^34,55^ Briefly, 3 μg total RNA extracted from SARS-CoV-2-infected Vero cells was treated with DNase I (NEB, M0303L) and followed by fragmentation as described above. The fragmented RNA was incubated with 8 μg anti-m^6^A antibody (Abcam, ab151230) in 450 μL immunoprecipitation buffer (100 mM NaCl, 50 mM Tris, pH 7.4, 0.05% NP-40) and incubated at 4 °C for about 2 h with rotating head over tail. The solution was then transferred to a clear and pre-cooled flat-bottom 24-well plate (Corning, 3524) on ice and irradiated twice with 0.15 J/cm^2^ UV light (254 nm) in a CL-1000 Ultraviolet Crosslinker (UVP). For immunoprecipitation, the mixture was collected and mixed with 40 μL pre-washed Dynabeads Protein A (Thermo Fisher Scientific, 1001D) at 4 °C for about 1.5 h. The beads–antibody–RNA mixture was then extensively washed and the PNK treatment (NEB, M0201S) was performed on beads for dephosphorylation. The m^6^A-marked RNA was eluted from beads by proteinase K (NEB, P8107S) digestion at 55 °C for 30 min followed by phenol–chloroform extraction and ethanol precipitation. The input and immunoprecipitated methylated RNA were subjected to library construction using SMARTer^®^ Stranded Total RNA-Seq Kit v2-Pico Input Mammalian (Takara, 634413) according to the manufacturer’s protocol. Sequencing was performed on Illumina Hiseq X Ten with paired-end 2 × 150 bp read length.

### Phylogenetic analysis

SARS-CoV-2 sequences published until 16th July were downloaded from GISAID. Sequences with no less than 29,000 bp length and no more than 5% (1500) unsolved nucleotides N were aligned by MAFFT.^37^ A total of 56,143 genome sequences of SARS-CoV-2 were selected for substitution analysis. The EPI_ISL_424359 (GWHACBB01000000) which was collected at early time in the pandemic was used as the reference sequence. 228 strains of viruses with substitution at m^6^A sites were detected. After removing highly similar sequences (similarity > 0.9998), 127 strains with substitution at the m^6^A methylation sites were selected. All of these 127 strains and 217 other virus sequences selected from 7 GISAID clade (L, S, G, GH, GR, O, V) with various collection locations and dates were used to construct maximum likelihood tree by IQ-TREE 2.^42^ The GTR + R2 substation model was evaluated as the best model from 286 candidates. Then the phylogenetic tree was constructed (rooted by EPI_ISL_424359) and the result was optimized by 1000 times bootstrap. The final result was visualized by ggtree.^56^

### Immunofluorescence assay

Cells were fixed with 4% (w/v) paraformaldehyde in PBS at RT for 15 min and blocked in PBS buffer containing 10% donkey serum and 0.3% Triton X-100 (Sigma, T8787) for 1 h at RT, followed by incubation with the primary antibodies at 4 °C overnight with 5% donkey serum and 0.15% Triton X-100. Nuclei were counterstained with DAPI DNA dye (CST, 4083, 1:1000) at RT for 10 min and mounted on glass slides. Images were taken using a PerkinElmer High Content Analysis System Operetta CLS and processed using Harmony 4.9 software. The following primary antibodies were used for immunofluorescence: anti-SARS-CoV-2 S protein (Sino Biological, Rabbit, T62, 1:500), anti-METTL14 and anti-ALKBH5 (Proteintech, 26158-1-AP and 16837-1-AP, Rabbit, 1:500).

### Quantitative analysis of m^6^A level

For the quantification of m^6^A level in uninfected and SARS-CoV-2-infected Vero and Huh7 cells, 75 ng purified RNA was digested into single nucleosides with 1 U nuclease P1 (Sigma, N8630) in 20 μL buffer containing 10 mM NH_4_Ac, pH 5.3 and incubated at 42 °C for 2 h. Subsequently, 1 U rSAP (NEB, M0371S) and 5 μL 0.5 M MES buffer (pH 6.5) were added and the mixture was incubated at 37 °C overnight. The digested RNA was injected into a LC-MS/MS which includes the ultra-performance liquid chromatography with a C18 column and the triple-quadrupole mass spectrometer (AB SCIEX QTRAP 6500). The positive ion multiple reaction-monitoring (MRM) mode was adopted to detect m^6^A abundance and m^6^A was quantified by the nucleoside to base ion mass transitions (282.0–150.1 for m^6^A and 268.0–136.0 for A). m^6^A levels in uninfected and SARS-CoV-2-infected Vero and Huh7 cells were calculated from the standard curve which was generated from pure nucleoside standards.

### Reads pre-processing and alignment

In our study, the strand orientation of the original RNA was preserved and sequences of reads 2 are sense to the original RNA. Thus, only reads 2 was used for m^6^A signal identification. Raw sequencing data was firstly subjected to Trim_galore (http://www.bioinformatics.babraham.ac.uk/projects/trim_galore/) for quality control and trimming adaptor. The quality threshold was set to 30, and the minimum length required for reads after trimming was 20 nt. The reads were then demultiplexed using fastq2collapse^39^ to remove PCR-amplified reads. Processed reads were mapped to genome (CoVID-19, Macaca, hg19, UCSC Genome Browser) using HISAT2 (version 2.1.0)^57^ with default parameters, and separated by strand with in-house scripts.

### Analysis of RNA-seq data

Adapter-clean reads were mapped to human and mouse genome (hg19, UCSC Genome Browser) using HISAT2 with default parameters. The expression of transcripts was quantified by FPKM using Cufflinks (version 2.2.1).^58^

### Identification of m^6^A peaks in SARS-CoV-2

Aligned and unique reads were subjected to exome-based peak caller exomPeak^59^ to detect significantly enriched m^6^A modification sites (FDR < 0.05) with default parameters. The number of reads in all input bam files was normalized to the same. MACS2 (version 2.1.1)^55^ was also used to identify m^6^A peaks, and the effective genome size was set to 2.7 × 10^9^ for human, 3.0 × 10^5^ for SARS-CoV-2 under the option of -*nomodel*. The *q*-value cutoffs were set to 0.01 for human and 0.05 for SARS-CoV-2, respectively. The reads coverage of peaks was showed by IGV (version 2.4.15)^60^ and RPKM was used as normalization method for comparison.

### m^6^A peak intensity

The m^6^A peak intensity was calculated as the ratio of RPKM_IP_/RPKM_input_ for each peak. The m^6^A peak from 24 h and 56 h samples were merged to generate the reference peak list. Peak intensity of 24 h and 56 h samples were calculated by reference peak.

### Analysis of miCLIP-seq data of SARS-CoV-2

miCLIP pipeline was used to identify m^6^A sites as previously described.^34^ Unique reads were subjected to downstream analysis. For each position, the unique reads cover (k) and the C-to-T transition reads (m) were counted, and known SNPs in the viral and Vero genome were removed. Then potential sites were filtered by both the number of C-to-T transitions (m) and the ratio of C-to-T transitions (m/k) of unique reads. Firstly, to avoid the mismatched sites caused by PCR amplification and library sequencing randomly, each transition had to be called at least twice for host (m > 2), and more than 50 times for SARS-CoV-2 (m > 50). To further improve the data credibility, the virus-unique reads coverage was required to be above 5000 (k > 5000). Secondly, the ratio of C-to-T transitions of unique reads were required ranging from 1% to 50% (1% < m/k < 50%), and the mismatches in viral genome of more than 0.3% in input sample were eliminated to reduce noise and simultaneously deplete sites with very high mismatch rates such as produced by SNPs and mapping artifacts. Additionally, the identified m^6^A sites located within the m^6^A peaks identified by RIP-seq will be considered highly confident.

### Motif discovery and GO enrichment analysis

To analyze sequence consensus, we chose the top 1000 peaks for de novo motif analysis with MEME (version 4.12.0),^61^ with 100-nt-long peak summit-centered sense sequences as input. Weblogo was used to analyze the sequence context of m^6^A sites.^62^ We performed Gene Ontology (GO) enrichment analyses using DAVID web-based tool (http://david.abcc.ncifcrf.gov/).^63^

### Correlation analysis of SNPs with m^6^A sites

To analyze the correlation between m^6^A sites and SNPs of SARS-CoV-2, upstream and downstream 800 bp was extended from m^6^A sites, and the 1600 bp region was divided into eight windows. The SNP database collected from China National Center for Bioinformation (https://bigd.big.ac.cn/ncov/) was intersected with m^6^A sites and extended windows using bedtools (version 2.27.1) to calculate the SNP frequency. Besides, 8 random sites (repeated 100 times) on the viral genome were selected as random backgrounds.

### Quantification and statistical analysis

*P* values were calculated using unpaired Student’s *t*-test and two-sided Mann–Whitney U-test. ****P < 0.0001; ***P < 0.001; **P < 0.01; * *P* < 0.05; ns, non-significant. Data were presented as means ± SD.

## Supplementary information

Supplementary Figure S1

Supplementary Figure S2

Supplementary Figure S3

Supplementary Figure S4

Supplementary Figure S5

Supplementary Figure S6

Supplementary Table S1

Supplementary Table S2

Supplementary Table S3

Supplementary Table S4

Supplementary Table S5

## Data Availability

The raw sequence data reported in this paper have been deposited in the Genome Sequence Archive in BIG Data Center, Beijing Institute of Genomics (BIG), Chinese Academy of Sciences, under accession number CRA002936 that is publicly accessible at http://bigd.big.ac.cn/gsa. All other data supporting the findings of this study are available from the corresponding author on reasonable request.

## References

[1] Cui J, Li F, Shi ZL (2019). Origin and evolution of pathogenic coronaviruses. Nat. Rev. Microbiol..

[2] Wu F (2020). A new coronavirus associated with human respiratory disease in China. Nature.

[3] Zhou P (2020). A pneumonia outbreak associated with a new coronavirus of probable bat origin. Nature.

[4] Kim D (2020). The architecture of SARS-CoV-2 transcriptome. Cell.

[5] Rehwinkel J (2010). RIG-I detects viral genomic RNA during negative-strand RNA virus infection. Cell.

[6] Wang Y (2015). Coronavirus nsp10/nsp16 methyltransferase can be targeted by nsp10-derived peptide in vitro and in vivo to reduce replication and pathogenesis. J. Virol..

[7] Machnicka MA (2013). MODOMICS: a database of RNA modification pathways–2013 update. Nucleic Acids Res.

[8] Desrosiers R, Friderici K, Rottman F (1974). Identification of methylated nucleosides in messenger RNA from Novikoff hepatoma cells. Proc. Natl. Acad. Sci. USA.

[9] Li X, Xiong X, Yi C (2016). Epitranscriptome sequencing technologies: decoding RNA modifications. Nat. Methods.

[10] Liu N, Pan T (2016). N6-methyladenosine-encoded epitranscriptomics. Nat. Struct. Mol. Biol..

[11] Fu Y, Dominissini D, Rechavi G, He C (2014). Gene expression regulation mediated through reversible m(6)A RNA methylation. Nat. Rev. Genet..

[12] Bokar JA, Rathshambaugh ME, Ludwiczak R, Narayan P, Rottman F (1994). Characterization and Partial-Purification of Messenger-RNA N-6-Adenosine Methyltransferase from Hela-Cell Nuclei - Internal Messenger-RNA Methylation Requires a Multisubunit Complex. J. Biol. Chem..

[13] Bokar JA, Shambaugh ME, Polayes D, Matera AG, Rottman FM (1997). Purification and cDNA cloning of the AdoMet-binding subunit of the human mRNA (N6-adenosine)-methyltransferase. RNA.

[14] Shi H, Wei J, He C (2019). Where, When, and How: context-dependent functions of RNA methylation writers, readers, and erasers. Mol. Cell.

[15] Jia G (2011). N6-methyladenosine in nuclear RNA is a major substrate of the obesity-associated FTO. Nat. Chem. Biol.

[16] Zheng G (2013). ALKBH5 is a mammalian RNA demethylase that impacts RNA metabolism and mouse fertility. Mol. Cell.

[17] Dominissini D (2012). Topology of the human and mouse m6A RNA methylomes revealed by m6A-seq. Nature.

[18] Meyer KD (2012). Comprehensive analysis of mRNA methylation reveals enrichment in 3’ UTRs and near stop codons. Cell.

[19] Zhou KI, Pan T (2018). An additional class of m(6)A readers. Nat. Cell Biol..

[20] Wang X (2014). N6-methyladenosine-dependent regulation of messenger RNA stability. Nature.

[21] Dominissini, D. & Rechavi, G. Epitranscriptome regulation. *Nat. Struct. Mol. Biol*. 10.1038/s41594-018-0140-7 (2018). 10.1038/s41594-018-0140-7 30266930

[22] Roundtree IA, Evans ME, Pan T, He C (2017). Dynamic RNA modifications in gene expression regulation. Cell.

[23] Song, J. & Yi, C. Reading chemical modifications in the transcriptome. *J. Mol. Biol*. 10.1016/j.jmb.2019.10.006 (2019). 10.1016/j.jmb.2019.10.006 31628951

[24] Frye M, Jaffrey SR, Pan T, Rechavi G, Suzuki T (2016). RNA modifications: what have we learned and where are we headed?. Nat. Rev. Genet..

[25] Dimock K, Stoltzfus CM (1977). Sequence specificity of internal methylation in B77 avian sarcoma virus RNA subunits. Biochemistry.

[26] Kane SE, Beemon K (1985). Precise localization of m6A in Rous sarcoma virus RNA reveals clustering of methylation sites: implications for RNA processing. Mol. Cell. Biol..

[27] Lavi S, Shatkin AJ (1975). Methylated Simian Virus 40-specific RNA from nuclei and cytoplasm of infected Bsc-1 cells. Proc. Natl. Acad. Sci. USA.

[28] Sommer S (1976). The methylation of adenovirus-specific nuclear and cytoplasmic RNA. Nucleic Acids Res..

[29] Lichinchi G (2016). Dynamics of human and viral RNA methylation during Zika virus infection. Cell Host Microbe.

[30] Tirumuru N. et al. N(6)-methyladenosine of HIV-1 RNA regulates viral infection and HIV-1 Gag protein expression. *Elife* **5**, e15528 (2016). 10.7554/eLife.15528 PMC4961459 27371828

[31] Lichinchi G (2016). Dynamics of the human and viral m(6)A RNA methylomes during HIV-1 infection of T cells. Nat. Microbiol..

[32] Gokhale NS (2016). N6-Methyladenosine in Flaviviridae viral RNA genomes regulates infection. Cell Host Microbe.

[33] Kennedy EM (2016). Posttranscriptional m(6)A editing of HIV-1 mRNAs enhances viral gene expression. Cell Host Microbe.

[34] Linder B (2015). Single-nucleotide-resolution mapping of m6A and m6Am throughout the transcriptome. Nat. Methods.

[35] Liu J (2020). Landscape and regulation of m(6)A and m(6)Am methylome across human and mouse tissues. Mol. Cell.

[36] Korber, B. et al. Tracking changes in SARS-CoV-2 Spike: evidence that D614G increases infectivity of the COVID-19 virus. *Cell* **182**, 812–827 (2020). 10.1016/j.cell.2020.06.043 PMC7332439 32697968

[37] Rozewicki J, Li S, Amada KM, Standley DM, Katoh K (2019). MAFFT-DASH: integrated protein sequence and structural alignment. Nucleic Acids Res..

[38] Cock PJA (2009). Biopython: freely available Python tools for computational molecular biology and bioinformatics. Bioinformatics.

[39] Harper JE, Miceli SM, Roberts RJ, Manley JL (1990). Sequence specificity of the human mRNA N6-adenosine methylase in vitro. Nucleic Acids Res..

[40] Wei CM, Moss B (1977). Nucleotide sequences at the N6-methyladenosine sites of HeLa cell messenger ribonucleic acid. Biochemistry.

[41] Wickham H. *ggplot2: elegant graphics for data analysis*. (Springer-Verlag, New York, 2016).

[42] Minh BQ (2020). IQ-TREE 2: new models and efficient methods for phylogenetic inference in the genomic era. Mol. Biol. Evol..

[43] Global Initiative on Sharing All Influenza Data (GISAID). Clade and lineage nomenclature aids in genomic epidemiology studies of active hCoV-19 viruses. https://www.gisaid.org/references/statements-clarifications/clade-and-lineage-nomenclature-aids-in-genomic-epidemiology-of-active-hcov-19-viruses/

[44] Sola I, Almazan F, Zuniga S, Enjuanes L (2015). Continuous and discontinuous RNA synthesis in coronaviruses. Annu. Rev. Virol..

[45] Khan S (2018). Comprehensive Review on Ebola (EBOV) Virus: future prospects. Infect. Disord. Drug Targets.

[46] Lu M (2020). N(6)-methyladenosine modification enables viral RNA to escape recognition by RNA sensor RIG-I. Nat. Microbiol.

[47] Chen YG (2019). N6-Methyladenosine modification controls circular RNA immunity. Mol. Cell.

[48] Lu W (2018). N(6)-Methyladenosine-binding proteins suppress HIV-1 infectivity and viral production. J. Biol. Chem..

[49] Shulman Z, Stern-Ginossar N (2020). The RNA modification N(6)-methyladenosine as a novel regulator of the immune system. Nat. Immunol..

[50] Mao Y (2019). m(6)A in mRNA coding regions promotes translation via the RNA helicase-containing YTHDC2. Nat. Commun..

[51] Aik W (2014). Structure of human RNA N(6)-methyladenine demethylase ALKBH5 provides insights into its mechanisms of nucleic acid recognition and demethylation. Nucleic Acids Res..

[52] Malacrida A (2020). 3D proteome-wide scale screening and activity evaluation of a new ALKBH5 inhibitor in U87 glioblastoma cell line. Bioorg. Med. Chem..

[53] Zeng Y (2018). Refined RIP-seq protocol for epitranscriptome analysis with low input materials. PLoS Biol..

[54] Sun H, Zhang M, Li K, Bai D, Yi C (2019). Cap-specific, terminal N(6)-methylation by a mammalian m(6)Am methyltransferase. Cell Res..

[55] Zhang C (2017). m(6)A modulates haematopoietic stem and progenitor cell specification. Nature.

[56] Yu GC, Smith DK, Zhu HC, Guan Y, Lam TTY (2017). GGTREE: an R package for visualization and annotation of phylogenetic trees with their covariates and other associated data. Methods Ecol. Evol..

[57] Kim D, Langmead B, Salzberg SL (2015). HISAT: a fast spliced aligner with low memory requirements. Nat. Methods.

[58] Trapnell C (2010). Transcript assembly and quantification by RNA-Seq reveals unannotated transcripts and isoform switching during cell differentiation. Nat. Biotechnol..

[59] Meng J, Cui X, Rao MK, Chen Y, Huang Y (2013). Exome-based analysis for RNA epigenome sequencing data. Bioinformatics.

[60] Thorvaldsdottir H, Robinson JT, Mesirov JP (2013). Integrative Genomics Viewer (IGV): high-performance genomics data visualization and exploration. Brief Bioinform..

[61] Bailey TL (2009). MEME SUITE: tools for motif discovery and searching. Nucleic Acids Res.

[62] Crooks GE, Hon G, Chandonia JM, Brenner SE (2004). WebLogo: a sequence logo generator. Genome Res..

[63] Huang DW, Sherman BT, Lempicki RA (2009). Systematic and integrative analysis of large gene lists using DAVID bioinformatics resources. Nat. Protoc..

