# Supplementary material for: The m6A methylome of SARS-CoV-2 in host cells
Source: Cell Res. 2021 Jan 28;31(4):404–14. doi: 10.1038/s41422-020-00465-7 (PMC8115241; doi:10.1038/s41422-020-00465-7)
Supplement: Supplementary file 1 — Supplementary Figure S1 [file 41422_2020_465_MOESM1_ESM.pdf]

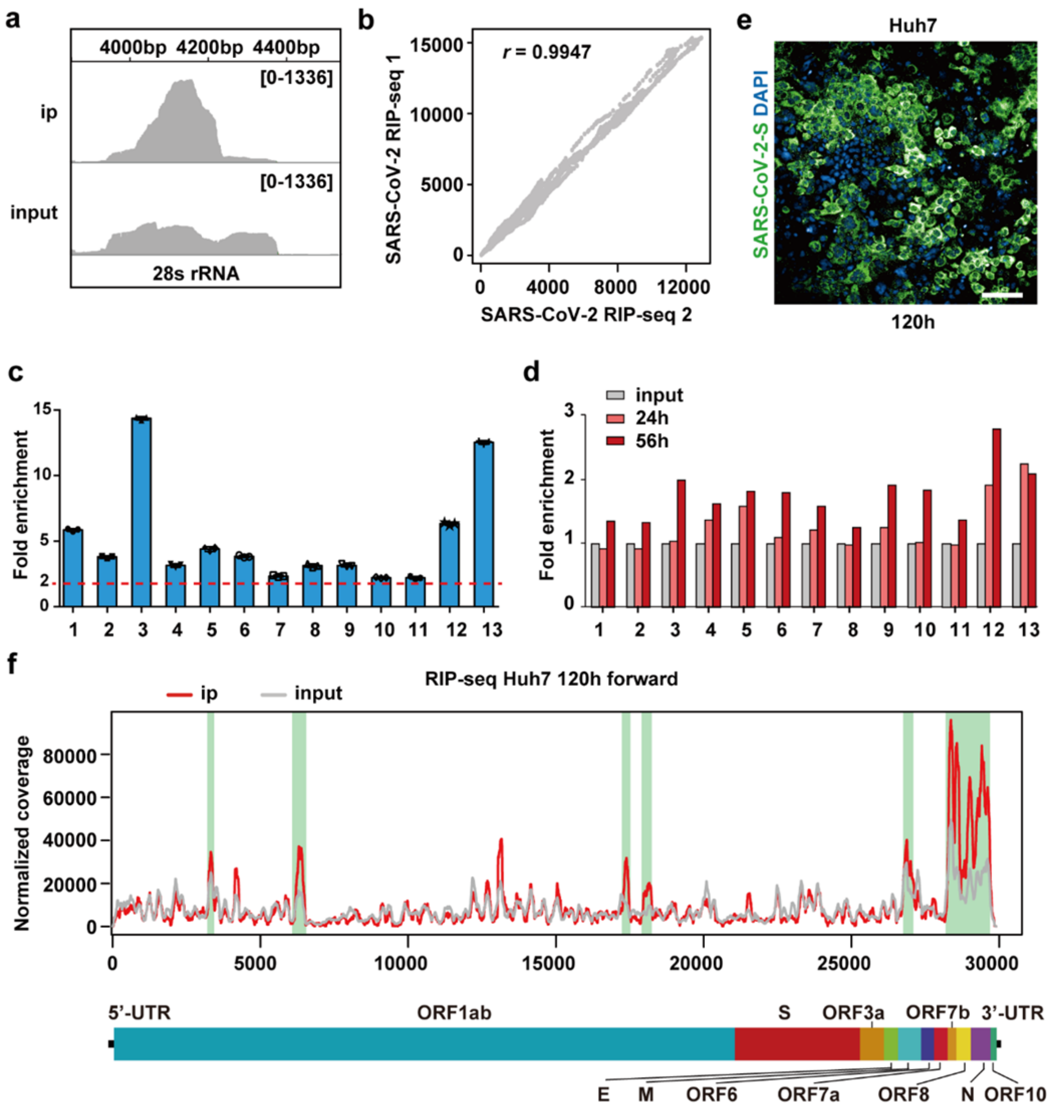

Fig S1. m^{6}A methylome of SARS-CoV-2 genomic RNA.

(**a**) IGV showing the RIP-seq reads of known m^{6}A residues at 4,190 position of 28S rRNA, suggesting good enrichment of RIP-seq.

(**b**) Correlation analysis of two biological replicates of SARS-CoV-2 RIP-seq data.

(**c**) Validation of the m^{6}A-marked viral RNA (13 m^{6}A peaks identified by RIP-seq) by performing m^{6}A-IP-qPCR using a different m^{6}A antibody (as an orthogonal evidence to the originally used Millipore m^{6}A antibody in RIP-seq) in Vero cells at 56 h post infection. Data are represented as mean ± SD; N = 3.

(**d**) m^6A peak intensity of SARS-CoV-2-infected Vero cells between 24h and 56h post infection. "peak intensity" is calculated as $RPKM_{IP}/RPKM_{Input}$ in each peak.

(**e**) Immunofluorescence of SARS-CoV-2-infected Huh7 cells (S protein, green) and nuclei (DAPI, blue) at 120h after infection.

(**f**) Refined RIP-seq of SARS-CoV-2 RNA harvested from Huh7 cells at 120 hpi showing the distribution of m^6A reads mapped to SARS-CoV-2 genome (red line). The baseline signal of input samples is represented by grey line and m^6A peaks are represented by green rectangles along the x axis. A schematic diagram of the SARS-CoV-2 genome is shown below to indicate the location of the m^6A-enriched sequences. Data are representative of N = 2 determinations.
