# Supplementary material for: The m6A methylome of SARS-CoV-2 in host cells
Source: Cell Res. 2021 Jan 28;31(4):404–14. doi: 10.1038/s41422-020-00465-7 (PMC8115241; doi:10.1038/s41422-020-00465-7)
Supplement: Supplementary file 2 — Supplementary Figure S2 [file 41422_2020_465_MOESM2_ESM.pdf]

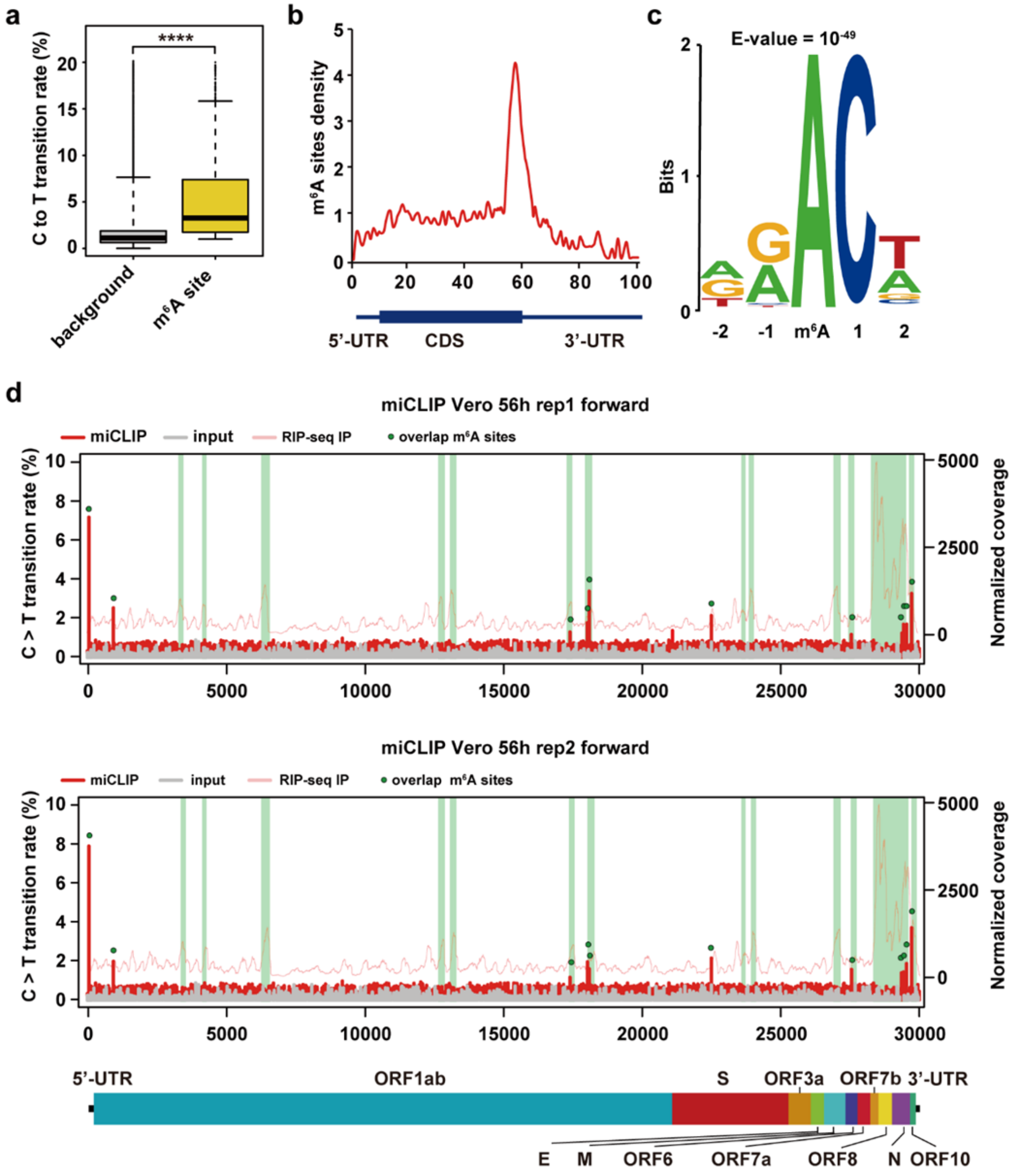

Fig S2. m^{6}A sites identified by miCLIP in Vero and SARS-CoV-2 genomic RNA.

(**a**) Boxplot showing the C to T transition rate of identified m^{6}A sites and background in host Vero cells. Statistical significance of the difference was determined by unpaired two-sided Mann-Whitney U-test. ****$P < 0.0001$.

(**b**) miCLIP-identified m^{6}A sites showed a metagene distribution profile typical for m^{6}A, suggesting good performance of miCLIP.

(**c**) Motif analysis revealed a ‘‘DRACH’’ consensus for m^6A sites identified by miCLIP in host Vero cells (E-value = 7.7×10^{-49}).

(**d**) m^6A sites at single-base resolution identified by miCLIP across SARS-CoV-2 genome. The grey line and red line showing the ratio of C to T transitions of input samples and immunoprecipitation samples, respectively. The green dots denote m^6A sites that located within the identified m^6A peaks (denoted by green rectangles) and are identified in both two biological replicates of miCLIP, while the grey dots showing the m^6A site identified only in miCLIP but not in the m^6A peaks identified by RIP-seq. The location of the m^6A-enriched sequences is shown by a schematic diagram of the SARS-CoV-2 genome.
