# Supplementary material for: The m6A methylome of SARS-CoV-2 in host cells
Source: Cell Res. 2021 Jan 28;31(4):404–14. doi: 10.1038/s41422-020-00465-7 (PMC8115241; doi:10.1038/s41422-020-00465-7)
Supplement: Supplementary file 3 — Supplementary Figure S3 [file 41422_2020_465_MOESM3_ESM.pdf]

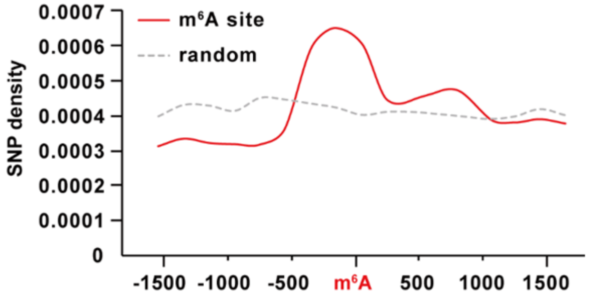

Fig S3. Correlation between SNPs and m^{6}A sites identified by miCLIP in SARS-CoV-2 genomic RNA.

SNP density analysis flanking the m^{6}A sites. SNP density of random background control is defined as 8 sites that are randomly sampled for 100 times. The SNP density in m^{6}A sites is significantly higher than that of random background ($P < 0.05$). Statistical significance of the difference was determined by unpaired two-sided Mann-Whitney U-test.
