# Supplementary material for: The m6A methylome of SARS-CoV-2 in host cells
Source: Cell Res. 2021 Jan 28;31(4):404–14. doi: 10.1038/s41422-020-00465-7 (PMC8115241; doi:10.1038/s41422-020-00465-7)
Supplement: Supplementary file 4 — Supplementary Figure S4 [file 41422_2020_465_MOESM4_ESM.pdf]

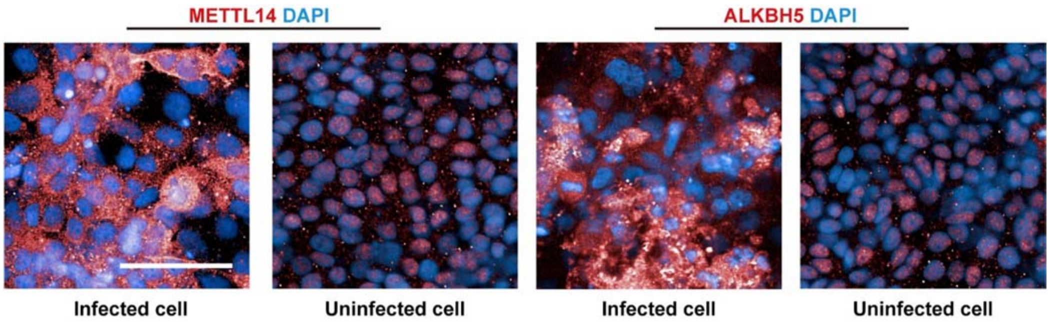

Fig S4. Immunostaining of METTL14 and ALKBH5 in SARS-CoV-2 infected or uninfected cells.

Nuclear and cytoplasmic localization of METTL14 methyltransferase and ALKBH5 demethylase visualized by immunostaining of uninfected and SARS-CoV-2-infected Huh7 cells at 72h after infection. Huh7 cells were infected by SARS-CoV-2 at 0.05 MOI for 72 hours and the uninfected group is shown as control. METTL14/ ALKBH5 is shown as red color and DAPI is represented by blue color. Scale bar, 20 μm.
