# Supplementary material for: The m6A methylome of SARS-CoV-2 in host cells
Source: Cell Res. 2021 Jan 28;31(4):404–14. doi: 10.1038/s41422-020-00465-7 (PMC8115241; doi:10.1038/s41422-020-00465-7)
Supplement: Supplementary file 5 — Supplementary Figure S5 [file 41422_2020_465_MOESM5_ESM.pdf]

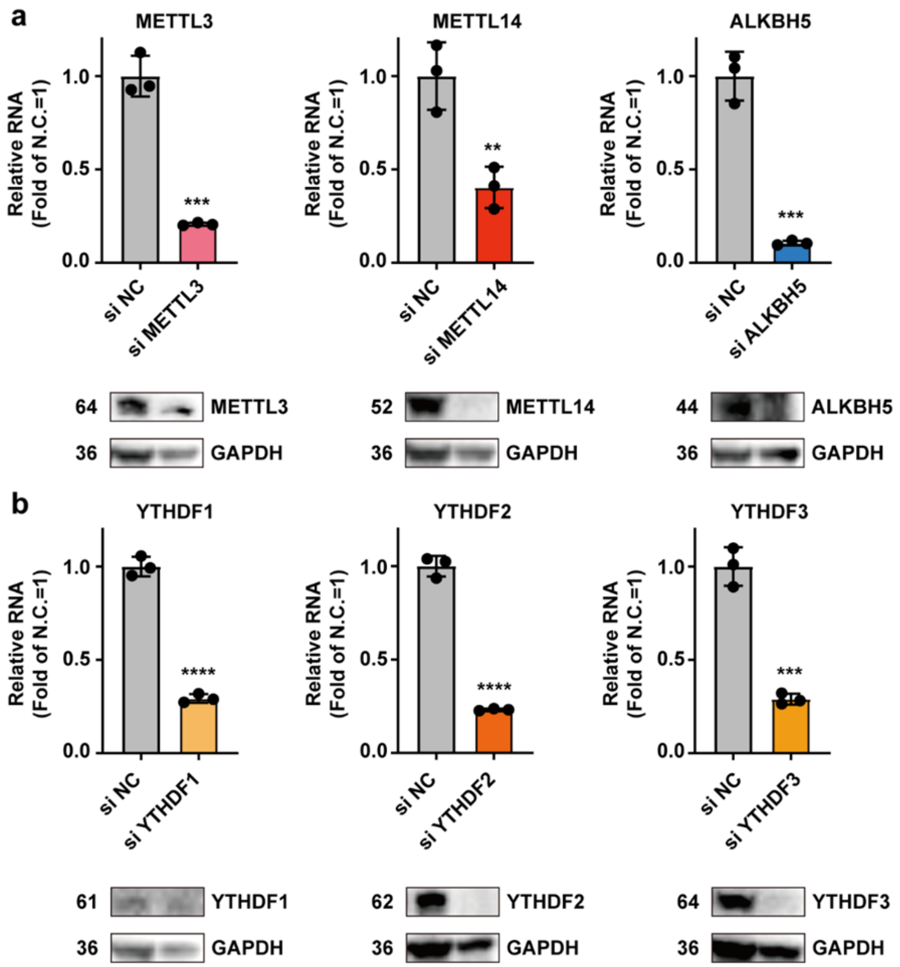

Fig S5. m^{6}A-related components knock down efficiency detection.

(**a**) Silencing efficiency of METTL3, METTL14, and ALKBH5 proteins in Huh7 cells expressing non-targeting shRNA (NTC) or the indicated gene-specific shRNAs, analyzed by qRT-PCR (top) or western blotting (bottom). All data are the mean ± SD of the indicated number of replicates (N = 3). Statistical significance of the difference was determined by unpaired Student's t-test. ****P* < 0.001; ***P* < 0.01.

(**b**) Silencing efficiency of m^{6}A reader proteins including YTHDF1, YTHDF2, and YTHDF3 in Huh7 cells expressing gene-specific shRNAs or non-targeting shRNA control (NTC) analyzed by qRT-PCR (top) or western blotting (bottom). All data are the mean ± SD of

the indicated number of replicates (N = 3). Statistical significance of the difference was determined by unpaired Student's t-test. ****$P < 0.0001$; ***$P < 0.001$.
