# Supplementary material for: The m6A methylome of SARS-CoV-2 in host cells
Source: Cell Res. 2021 Jan 28;31(4):404–14. doi: 10.1038/s41422-020-00465-7 (PMC8115241; doi:10.1038/s41422-020-00465-7)
Supplement: Supplementary file 6 — Supplementary Figure S6 [file 41422_2020_465_MOESM6_ESM.pdf]

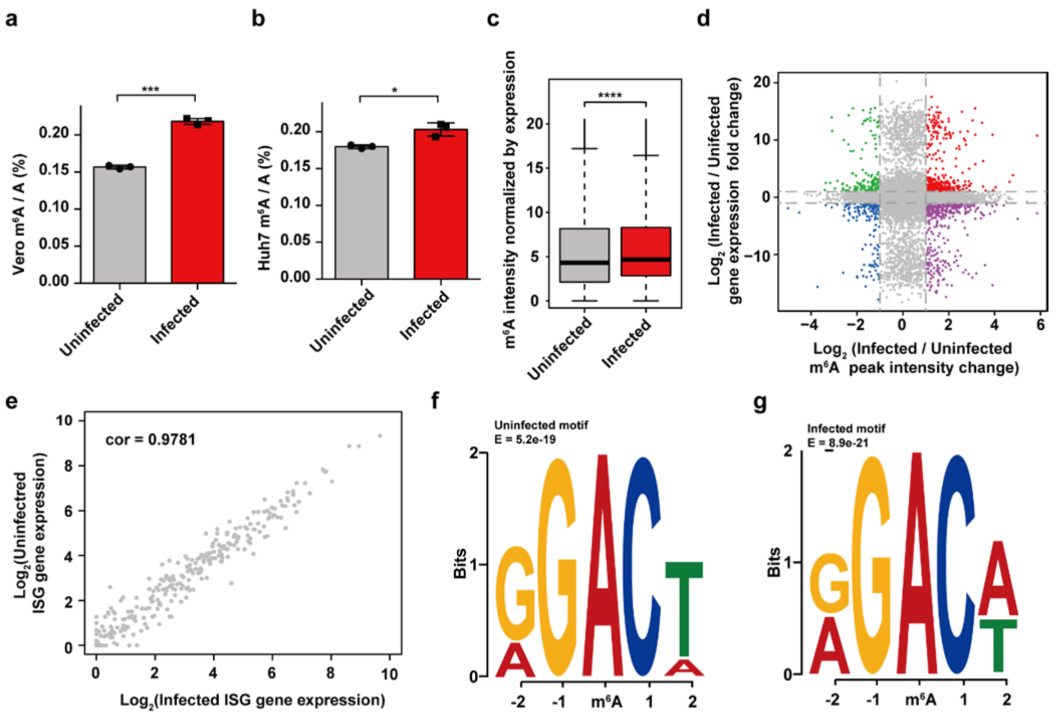

Fig S6. The influences of SARS-CoV-2 infection on m^{6}A methylome of Huh7 cell transcripts.

(**a and b**) m^{6}A abundance quantification in uninfected and SARS-CoV-2-infected Vero (**a**) and Huh7 cells (**b**) by the liquid chromatography-tandem mass spectrometry (LC-MS/MS). All data are the mean ± SD of the indicated number of replicates (N = 3). Statistical significance of the difference was determined by unpaired Student's t-test. ***$P < 0.001$; *$P < 0.05$.

(**c**) m^{6}A intensity analysis in uninfected and SARS-CoV-2-infected Huh7 cells. Statistical significance of the difference was determined by unpaired two-sided Mann-Whitney U-test. ****$P < 0.0001$.

(**d**) Correlation between changes in gene expression and m^{6}A modification between uninfected and SARS-CoV-2-infected Huh7 cells. Red dots showing transcripts with

increased gene expression level and upregulated m^6A signals; pink dots showing transcripts with decreased gene expression level and upregulated m^6A signals; green dots showing transcripts with increased gene expression level and downregulated m^6A signals; blue dots showing transcripts with decreased gene expression level and downregulated m^6A signals; grey dots showing transcripts with either unchanged gene expression level or unchanged m^6A signals.

(**e**) Correlation analysis of expression level of interferon-stimulated genes between uninfected and SARS-CoV-2-infected Huh7 cells.

(**f and g**) Transcriptome-wide motif analysis revealed consensus m^6A peaks in uninfected (**f**) and SARS-CoV-2-infected Huh7 cells (**g**), respectively.
